# Anesthesiologists' practice patterns for treatment of postoperative nausea and vomiting in the ambulatory Post Anesthesia Care Unit

**DOI:** 10.1186/1471-2253-6-6

**Published:** 2006-06-01

**Authors:** Alex Macario, Louis Claybon, Joseph V Pergolizzi

**Affiliations:** 1 Department of Anesthesia, and Health Research & Policy, Stanford University School of Medicine, Stanford, CA, USA; 2 Mercy Hospital Anderson, Cincinnati, Ohio, USA; 3 Johns Hopkins University School of Medicine, Baltimore, MD, USA

## Abstract

**Background:**

When patients are asked what they find most anxiety provoking about having surgery, the top concerns almost always include postoperative nausea and vomiting (PONV). Only until recently have there been any published recommendations, mostly derived from expert opinion, as to which regimens to use once a patient develops PONV. The goal of this study was to assess the responses to a written survey to address the following questions: 1) If no prophylaxis is administered to an ambulatory patient, what agent do anesthesiologists use for treatment of PONV in the ambulatory Post-Anesthesia Care Unit (PACU)?; 2) Do anesthesiologists use non-pharmacologic interventions for PONV treatment?; and 3) If a PONV prophylaxis agent is administered during the anesthetic, do anesthesiologists choose an antiemetic in a different class for treatment?

**Methods:**

A questionnaire with five short hypothetical clinical vignettes was mailed to 300 randomly selected USA anesthesiologists. The types of pharmacological and nonpharmacological interventions for PONV treatment were analyzed.

**Results:**

The questionnaire was completed by 106 anesthesiologists (38% response rate), who reported that on average 52% of their practice was ambulatory. If a patient develops PONV and received no prophylaxis, 67% (95% CI, 62% – 79%) of anesthesiologists reported they would administer a 5-HT3-antagonist as first choice for treatment, with metoclopramide and dexamethasone being the next two most common choices. 65% (95% CI, 55% – 74%) of anesthesiologists reported they would also use non-pharmacologic interventions to treat PONV in the PACU, with an IV fluid bolus or nasal cannula oxygen being the most common. When PONV prophylaxis was given during the anesthetic, the preferred PONV treatment choice changed. Whereas 3%–7% of anesthesiologists would repeat dose metoclopramide, dexamethasone, or droperidol, 26% (95% confidence intervals, 18% – 36%) of practitioners would re-dose the 5-HT3-antagonist for PONV treatment.

**Conclusion:**

5-HT3-antagonists are the most common choice for treatment of established PONV for outpatients when no prophylaxis is used, and also following prophylactic regimens that include a 5HT3 antagonist, regardless of the number of prophylactic antiemetics given. Whereas 3% – 7% of anesthesiologists would repeat dose metoclopramide, dexamethasone, or droperidol, 26% of practitioners would re-dose the 5-HT3-antagonist for PONV treatment.

## Background

When patients are asked what they find most anxiety provoking about having surgery, the top concerns almost always include postoperative nausea and vomiting (PONV) [1,2]. Anesthesiologists agree that PONV is an important issue for patients [3]. Since PONV is important to patients, improving the quality of anesthesia care includes reducing the incidence and severity of PONV. A large number of prospective randomized clinical trials have been completed to evaluate the efficacy of drugs and non-pharmacologic interventions to prevent PONV [4-8]. Data also exist on what prophylaxis interventions anesthesiologists in routine clinical practice actually administer for PONV [9].

However, fewer studies investigate the efficacy of antiemetics for the treatment of PONV once it occurs in the Post Anesthesia Care Unit (PACU). For example, a quantitative systematic review of treatment of established PONV published in 2001 found that metoclopramide, droperidol, isopropyl alcohol vapor, and midazolam were tested in one trial only, each with a limited number of patients [10]. That review also found that 5-HT3 antagonists had absolute risk reductions compared with placebo of 20% – 30%, with a less pronounced anti-nausea effect.

The discrepancy between the plethora of trials on prevention of PONV and the paucity of trials on treatment of established symptoms is due, in part, to the difficulty in performing PONV treatment studies since a large number of patients would be needed to obtain the required target sample size that eventually experience PONV. In fact, only until recently, have there been any published recommendations, mostly derived from expert opinion not clinical trials, as to which regimens to use once a patient develops PONV [11].

Few USA data exist on practice patterns for treatment of PONV once it occurs in the PACU. PONV treatment data could be collected prospectively or abstracted retrospectively from the anesthesia and medical record. However, these methods make it difficult to compare practice patterns among practitioners, as neither method controls for differences in patient's severity of illness, demographics, or practice type. Other disadvantages of the chart review methodology include recording bias (e.g., some interventions may be provided but not documented) and the skilled (and costly) experts required to accurately collect data from the medical record.

To isolate physician practice from confounding variables, simple case vignettes have been validated as a method to elicit medical practice treatment patterns [12]. Vignettes are written cases that simulate actual clinical practice. Educators, demographers, and health service researchers have used these vignettes to measure processes in a wide range of settings [13-15].

The goal of this study was to assess the responses to a written questionnaire (with short hypothetical clinical vignettes) to address the following questions regarding PONV in the PACU: 1) If no prophylaxis is administered to an ambulatory patient, what agent do anesthesiologists use for treatment?; 2) Do anesthesiologists use non-pharmacologic interventions for PONV treatment?; and 3) If a PONV prophylaxis agent is administered during the anesthetic, do anesthesiologists choose an antiemetic in a different class for treatment?

## Methods

Approval for this study was obtained from the Stanford University Human Subjects Committee.

### Physician sample

We mailed a written questionnaire to 300 anesthesiologists selected at random from the 2002 American Society of Anesthesiology Directory available to us in printed form. A random number generator was used to select names, the number indicating how far down the list to go on each page. We chose 300 because based on previous studies, we expected approximately a 33% response rate, which would generate our goal of 100 surveys returned. Questionnaires were sent by U.S. mail to each subject's address during December 2004. A stamped self-addressed envelope was to enhance the response rate.

### Survey measurement methods

The survey instrument consisted of three parts. The first page was a cover letter, the second page requested basic demographic data about the respondent, and the third page contained the clinical scenarios or vignettes. [see Additional file 1] For example, the stem, or base case, for vignette #1 was, "A 22-yr-old woman status post outpatient pelvic laparoscopy under general anesthesia. She received no PONV prophylaxis. In the PACU, she reports PONV. What would your antiemetic order(s) be?"

Questionnaire instructions included: "Assume all other relevant clinical history and exam is negative. Assume patients have received adequate analgesics." We did not specify whether responders could use monotherapy or combination therapy.

To assess how prophylaxis choice affected treatment, the above vignette stem stayed the same for vignettes #2, #3, #4 and #5, but the number of prophylaxis anti-emetics increased from one to four. Vignette # 2 had the patient receive a 5-HT3 anatagonist for prophylaxis, vignette # 3 had a 5-HT3 antagonist and metoclopromide for prophylaxis, and vignette #4 had a 5-HT3 antagonist, metoclopromide and dexamethasone. Vignette #5 stated, "A 22-yr-old woman status post outpatient pelvic laparoscopy under general anesthesia. She received a 5-HT3 antagonist, metoclopromide, dexamethasone, and droperidol for prophylaxis. In the PACU, she reports PONV. What would your antiemetic order(s) be?"

We also aimed to assess the second choice for treatment if initial PONV treatment fails. For each vignette we asked, "What is your second choice for treatment if the first treatment fails?"

Four senior, board-certified anesthesiologists in the Stanford Department of Anesthesiology reviewed the vignettes to ensure adequate content.

Five other anesthesiologists (convenience sample) were asked to take the questionnaire twice, two days apart, in a non-random, non-anonymous fashion to assist with checking the internal reliability of the questionnaire. All five respondents answered every question. Of 90 eligible responses (demographic questions were excluded), 82% were answered the same way the second time.

95% two-sided confidence intervals were calculated for N1/(N1+N2) using Clopper-Pearson method (StatXact-6, Cytel Software Corporation, Cambridge, MA) [16].

## Results

Of the 300 questionnaires mailed out, twenty-one were returned as undeliverable because the person was no longer at that address, and three were returned with the respondent stating they were no longer in active clinical practice. The 106 completed questionnaires returned gave a response rate of 106/276 or 38%. (see Table 1 for demographics)

**Table 1:** Demographics of respondents (N = 106)

Mean years in practice (SD, range)	19 (8, 3 – 41)
Mean age (SD, range)	47 (8, 31 – 68)
Primary practice location	
Hospital-Inpatient	65%
Hospital-Outpatient	16%
Free-standing surgery center	17%
Surgeon office	1%
Male	85%
Practice Characteristics	
Academic	43%
Private Practice	57%
% of practice that is ambulatory	52%
Number of states represented	19

Sixty-seven % (95% confidence intervals, 62% – 79%) of the anesthesiologists we surveyed reported they would administer a 5-HT3 antagonist as first choice if no prophylaxis had been administered. (Table 2) (Ondansetron (53%) + dolasetron (13%) + granisetron (1%) = 67%)

**Table 2:** Initial treatment for PONV by the prophylaxis agent given

	Vignettes with different prophylaxis regimens				
	
	None	5-HT3	5-HT3 & meto	5-HT3 & meto & dexa	5-HT3 & meto & dexa & drop
Pharmacologic treatment:					
Ondansetron	53%	22%	23%	23%	23%
Dolasetron	13%	3%	2%	5%	4%
Droperidol	7%	14%	18%	19%	7%
Dexamethasone	8%	15%	19%	2%	3%
Metoclopramide	11%	21%	4%	5%	4%
Promethazine	3%	13%	20%	22%	28%
Prochlorperazine	0%	1%	1%	2%	4%
Diphenhydramine	0%	0%	0%	5%	4%
Intra-muscular ephedrine	1%	4%	4%	5%	5%
Hydroxyzine	1%	2%	2%	5%	4%
Propofol	0%	1%	1%	2%	5%
Scopolamine patch	1%	1%	1%	2%	3%
Granisetron	1%	1%	1%	1%	1%
Other	1%	2%	2%	2%	3%
Total	100%	100%	100%	100%	100%
Other includes: trimethobenzamide, perphenazine, haloperidol, atropine, or midazolam					
5-HT3 = 5-HT3-antagonist; meto = metoclopramide; dexa = dexamethasone; drop = droperidol					
Non-pharmacologic treatment:					
IV fluid bolus	57%	55%	56%	57%	51%
Oxygen nasal cannula	22%	19%	19%	19%	20%
Sniff alcohol swab	6%	5%	7%	7%	6%
Reassure the patient that PONV will pass	1%	3%	1%	1%	1%
Forced air warming	3%	4%	3%	3%	7%
Keep NPO	1%	1%	3%	1%	1%
Acupressure forearm & acupuncture & acustimulation with the ReliefBand	4%	4%	4%	4%	6%
Lay patient flat on gurney	4%	3%	3%	3%	3%
Other	0%	5%	3%	3%	4%
Total	100%	100%	100%	100%	100%

Other includes: encourage emptying of oropharynx/spitting, assure there is no bleeding, transfer to inpatient ward, or add glucose to IV

Metoclopramide, with 11% (95% confidence intervals, 6.3% – 18%) of anesthesiologists choosing as first option, and dexamethasone (8% of anesthesiologists, 95% confidence intervals, 3.3% – 14%) were the next two most popular agents for PONV treatment when no prophylaxis had been given.

Sixty-five % (95% confidence intervals, 55% – 74%) of anesthesiologists would use non-pharmacologic interventions for treatment. An IV fluid bolus or oxygen via nasal cannula were the two most common choices. (Table 2)

PONV treatment choice changed depending on prophylaxis agent given. (Table 2) For example, only approximately 5% of anesthesiologists reported they would repeat dose the metoclopramide, approximately 3% would repeat the dexamethasone, and 7% would repeat the droperidol. In contrast, when a 5-HT3 antagonist was used for monotherapy prophylaxis, a repeat dose of the 5-HT3 antagonist was administered by 26% (95% confidence intervals, 18% – 36%) of survey responders. Promethazine utilization increased as a treatment choice as the number of other drugs (the 5-HT3 antagonist, metoclopramide, dexamethasone, and droperidol) for prophylaxis increased as stated in our vignettes. (Table 2)

If no prophylaxis was administered and initial therapy for PONV failed, then the most common (reported by 24% of anesthesiologists) next choice for treatment was still a 5-HT3 antagonist, followed by promethazine.

Thirty-seven % of respondents wrote in some free text under the comments section. Forty-four % of the comments explained or reinforced answers given in the main part of the questionnaire, while 33% of the written-in comments related to droperidol availability and the FDA Black Box warning. The remaining 23% of comments referred to a variety of issues such as: formulary availability, "I examine every patient. What I do depends on my exam," "Need more treatment studies," and "Treatment and choice are often driven by the nursing staff."

## Discussion

Our written questionnaire study found that 5-HT3-antagonists are the most common choice for treatment of established PONV when no prophylaxis is used. This pattern holds true following PONV prophylaxis with a regimen including a 5 HT3 antagonist regardless of the number of prophylactic antiemetics received by the patient

Overall, anesthesiologists reported administering a total of eighteen different drugs for PONV treatment and twelve different non-pharmacologic interventions. Variations in medical practice such that physicians treat similar patients differently may be created by uncertainty about efficacy of interventions, or formulary restrictions, or recent visits by the pharmaceutical sales representative, as well as differences in practitioners' residency training, judgment, and beliefs about drug acquisition costs and side-effect profiles [17-19]. Enhanced education and individualized feedback can change anesthesiologists' practice patterns [20,21].

### Initial treatment

According to responses to a specific question on the questionnaire, almost all anesthesiologists (96%) preferred pharmacologic interventions for treatment, instead of non-pharmacologic (e.g., hydration, oxygen, acupuncture). The four anti-emetics chosen to be included for prophylaxis in the hypothetical patient vignettes – a 5-HT3 antagonist, droperidol, dexamethasone, and metoclopramide – were intended to represent major receptor systems involved in the etiology of PONV, as well as agents commonly used in clinical practice. We did not specify the doses of the four chosen antiemetics because we were mainly interested in the choice not the dose. To keep survey length reasonable, we opted not have a vignette with promethazine as a prophylaxis agent because in our outpatient practice promethazine is infrequently given for prevention.

Two-thirds of the anesthesiologists reported they would administer a 5-HT3 antagonist as first choice for PONV treatment if no prophylaxis had been given. The efficacy of the 5-HT_3 _antagonists may be more pronounced when a patient is vomiting than as treatment for nausea. There is weak evidence of dose-responsiveness with these drugs [22,23]. Therefore, small doses of the 5-HT_3 _antagonists (ondansetron 1 mg) have been recommended for treatment. Interestingly, less than 15% of anesthesiologists reported using a combination of several agents for treatment, despite that combination of agents, or multi-modal therapy, may be increasingly being used for prophylaxis.

### Repeat dosing

A majority of anesthesiologists reported they changed to a different agent for PONV treatment than the one(s) used for prophylaxis. However, 26% of practitioners would administer a second dose of the 5HT-3 antagonist (ondansetron (22%) + dolasetron (3%) + granisetron (1%)) if initial 5HT-3 antagonist prophylaxis failed. This is despite consensus guidelines, mostly derived from expert opinion not clinical trials, which suggest that if PONV occurs within six hours postoperatively, patients should not receive a repeat dose of the prophylactic antiemetic. Prescribing information for ondansetron states that a second dose does not provide additional control if the first prophylactic dose has failed. A drug from a different class should be used for treatment [24].

Pharmacogenomics may affect the success of a 5-HT3 antagonist because some patients have extra copies of the CYP2D6 gene, a genotype consistent with ultrarapid metabolism [25]. A separate study of patients who failed prophylaxis with ondansetron found the complete response rate was significantly higher after treatment with promethazine (78%) than after treatment with repeat ondansetron (46%) [26]. A third study of 428 patients (of 2,199 prophylactically treated with ondansetron) with PONV in the PACU, found that an additional dose of ondansetron was no better than placebo for reducing PONV two hours postoperatively [27].

Interestingly, anesthesiologists in our survey study were less likely to redose metoclopramide, dexamethasone, or droperidol for treatment (than a 5-HT3 antagonist) if any of those agents were administered for prophylaxis.

One quarter of anesthesiologists reported not having preprinted PACU orders specifically for PONV. This may increase the variability in PONV clinical practice, and make it difficult for evidence-based care to be implemented. Better mechanisms for delivering decision clinical support (e.g., evidence based guidelines) for PONV in the PACU may be possible. Four % of our sample voluntarily indicated that their group had developed their own PONV treatment guidelines.

For "older generation" antiemetics there are few data on therapeutic efficacy for established PONV. As an example, in patients who failed prophylaxis with droperidol, the complete response rate was significantly higher after treatment with promethazine (77%) than after droperidol (56%) [26].

It may be that anesthesiologists believe that interventions shown to be effective for prevention will be similarly effective for treatment. For example, many of our responders indicated they would use supplemental oxygen to treat PONV, but most studies of oxygen have been for PONV prevention, with varying efficacy [28,29]. Other non-pharmacologic treatments suggested by our respondents such as IV fluid therapy, isopropyl alcohol inhalation and acupuncture/acustimulation have been studied, sometimes for prophylaxis not treatment, while others such as forced air warming have not [30,31].

Beyond six hours, PONV can be treated a second time with any of the agents used for prophylaxis except dexamethasone and scopolamine, which are longer acting. We found that 73% of anesthesiologists reported having preprinted PACU orders for PONV at their primary practice location such that the anesthesiologist can amend the orders via checkbox, or by writing in.

To keep the questionnaire a reasonable length, we did not ask respondents why they chose the different treatments. The next study will assess if choices are based on such items as department policy, cost considerations, perceived lack of evidence or insufficient knowledge on the part of the anesthesiologist, individual patient's condition, or nursing determination.

### PONV treatment research requires more precise PONV assessment

The lack of consistent assessment of PONV is an issue because studies often define endpoints differently. Nausea sometimes is defined by patient self-report, and other times as an observer asking the patient a yes/no answer. Some institutions define PONV as when actual treatment of PONV occurs, which is easily quantified, but is confounded because patients' perceptions of nausea severe enough to require intervention varies among patients, and nurses have different thresholds for initiating treatment [32]. Often nausea and vomiting are not distinguished and the symptoms of PONV are combined into a single PONV endpoint [33]. The challenge of multiple end-points and heterogeneity of definitions need to be addressed before aiming to establish the optimal management of PONV once it occurs in the PACU. The entire observation period should cover 24 hrs [34]. Treatment responses we obtained might have varied between a patient developing nausea alone or having vomiting.

### Limitations

To control for the potential impact of biases from differing case-mix, we employed a postal questionnaire vignette methodology. The limitations of this method include that the subject sample depended on anesthesiologists' willingness to participate [35]. While not significantly different from other national surveys of professional organizations, the response rate of 38 % is low and non-response bias may exist. This bias reflects the fraction of eligible subjects that do not respond and the difference in their answers compared to responders. Since it is unknown whether the physicians answering the questionnaire were systematically different from non-responders, there is no absolutely acceptable level of response.

The study had relatively small sample size. Determination of adequate sample size may be difficult and depends on the desired precision of the results. A larger number of respondents is always possible (to enable subgroup analyses about differences among practice types, academic vs. private practice, for example) but we obtained a reasonable sampling of current practice patterns to help design larger studies of PONV treatment.

Also, our result that a 5-HT3 antagonist is the most commonly prescribed for PONV treatment may not be applicable in other countries.

Although vignettes are suitable for comparative analyses because they control for case-mix, further studies are needed to confirm that the results from vignette-based questionnaires are in fact a valid measure of the real-life clinical care provided by anesthesiologists. The open-ended comments section in our questionnaire did not uncover any problems with anesthesiologists stating they didn't understand the questionnaire, or that key elements were missing. Since our vignettes were hypothetical, the answers provided by the anesthesiologists may not be what they actually use.

## Conclusion

5-HT3-antagonists are the most common choice for treatment of established PONV for outpatients when no prophylaxis is used, and also following prophylactic regimens that include a 5HT3 antagonist, regardless of the number of prophylactic antiemetics given. Whereas 3%–7% of anesthesiologists would repeat dose metoclopramide, dexamethasone, or droperidol, 26% of practitioners would re-dose the 5-HT3-antagonist for PONV treatment. PONV guidelines may help reduce this unnecessary redosing.

## Competing interests

This study was funded in part by GlaxoSimthKline. GlaxoSimthKline did not participate in any of the following: study design, questionnaire development, data collection, analysis, interpretation of the data, writing of the manuscript, or decision to submit the manuscript for publication.

## Authors' contributions

AM conceived and designed the study, led the data analysis, and wrote the manuscript.

LC and JP participated in the study's design, data analysis and revised the manuscript.

All authors read and approved the final manuscript.

## Pre-publication history

The pre-publication history for this paper can be accessed here:



## Supplementary Material

Additional file 1 Survey instrument A short description of the data: Survey instrument with questions and vignettes Click here for file
