# Supplementary material for: Anesthesiologists' practice patterns for treatment of postoperative nausea and vomiting in the ambulatory Post Anesthesia Care Unit
Source: BMC Anesthesiol. 2006 Jun 1;6:6. doi: 10.1186/1471-2253-6-6 (PMC1525160; doi:10.1186/1471-2253-6-6)
Supplement: Additional file 1 — Survey instrument A short description of the data: Survey instrument with questions and vignettes [file 1471-2253-6-6-S1.pdf]

Survey instrument

Dear Colleague,

Hello, my name is Alex Macario. I am on the faculty at Stanford. I am writing to ask for your help in participating in a research study, by completing a written questionnaire. You were randomly selected to participate from the American Society of Anesthesiologists directory.

I am surveying how anesthesiologists treat postoperative nausea and vomiting (PONV) once PONV occurs in the recovery room after outpatient surgery. Very little is published on this issue.

This survey does not ask about PONV prophylaxis, but rather treatments you order once a patient reports having PONV.

The reason I am interested in this topic is that these data on practice patterns may be useful for understanding how to optimize the decision to provide PONV treatment.

Please complete the enclosed questionnaire and return it in the envelope provided. It should take approximately 15 minutes to fill out. No payment will be provided to you nor will you receive any other direct benefits. Your participation is entirely voluntary.

Do not put your name on the survey. Your answers are completely anonymous (no one will be able to identify this one as yours). Your return of the completed survey implies consent.

We are not requesting that you sign and return a consent form. This is because the research involves minimal risk to you. Also, we believe the anonymity of the responses will lead to more accurate responses. You have the right to discontinue participation at any time without penalty, and you have the right to withdraw consent. Please keep a copy of this cover letter for your records. You have the right to refuse to answer particular questions.

If you have any questions about this study, please contact Dr. Alex Macario at (650) 723-6411. For further information about your rights as a research subject or if you are not satisfied with the manner in which this study is being conducted, you may contact (anonymously if you wish): the Human Subjects Office, Stanford University, Stanford, CA 94305-5401, or by phone (650) 723-4697.

Thank you for your help.

Alex Macario

Please complete this questionnaire and return it in the addressed, stamped, enclosed envelope. The purpose of this study is to survey current clinical practices for treatment of PONV in outpatients.

1. Your age: ________ yrs

2. Gender: M ____ F ____

3. What is your current status (circle one please): Academic Private Other ______

4. Years in clinical anesthesia practice (including residency): _______ yrs

5. What % of your practice involves ambulatory surgery (same day surgery/discharge)? ___ %

6. Do you supervise nurse anesthetists? Yes_____ No______

7. What is the primary location of your practice? {Please choose one answer}
Hospital-Inpatient Services Hospital-Outpatient Free-standing surgery center Physician office

8. Do you have preprinted PONV orders for PACU? Yes__ No___ If yes, which one below is true?

__ PONV orders are pre-written such that every patient receives the same orders
__ PONV orders are pre-written such that the anesthetist can amend the orders via checkbox, or writing in

---

Assume the hypothetical ambulatory patients described below report PONV in the PACU. Please write in what your interventions would be to treat PONV. Assume all other relevant clinical history and exam is negative. Assume patients have received adequate analgesics.

Patient # 1 – a 22 yr old woman s/p outpatient pelvic laparoscopy under general anesthesia. She received no PONV prophylaxis. In the PACU, she reports PONV. What would your initial antiemetic order(s) be (please include dosage)?

Pharmaacologic: ___________________________________
Non-pharmacologic: __________________________________

What is your 2nd choice for treatment if the first treatment fails? __________________

Patient # 2 – a 22 yr old woman s/p outpatient pelvic laparoscopy under general anesthesia. She received a serotonin 5-HT3 antagonist (the one available at your hospital) for PONV prophylaxis. In the PACU, she reports PONV. What would your antiemetic order(s) be (include dosage)?

Pharmacologic: _____________________________________
Nonpharmacologic: _____________________________________

What is your 2nd choice for treatment if the first treatment fails?_____________

Patient # 3 – a 22 yr old woman s/p outpatient pelvic laparoscopy under general anesthesia. She received a 5-HT3 antagonist and metoclopromide for PONV prophylaxis. In the PACU, she reports PONV. What would your antiemetic order(s) be (include dosage)?

Pharmacologic: ______________________________________
Nonpharmacologic: ______________________________________

What is your 2nd choice for treatment if the first treatment fails?_____________

Patient # 4 – a 22 yr old woman s/p outpatient pelvic laparoscopy under general anesthesia. She received a 5-HT3 antagonist, metoclopromide, and dexamethasone for PONV prophylaxis. In the PACU, she reports PONV. What would your antiemetic order(s) be?

Pharmacologic: ______________________________________
Nonpharmacologic: ______________________________________

What is your 2nd choice for treatment if the first treatment fails?_____________

Patient # 5 – a 22 yr old woman s/p outpatient pelvic laparoscopy under general anesthesia. She received 5-HT3 antagonist, metoclopromide, dexamethasone, & droperidol for prophylaxis. In the PACU, she reports PONV. What would your antiemetic order(s) be (please include dosage)?

Pharmacologic: ______________________________________
Nonpharmacologic: ______________________________________

What is your 2nd choice for treatment if the first treatment fails?_____________

In general, what is your first preference? (please circle one)

pharmacologic   non-pharmacologic   no preference of one over the other

Please list any comments. Thank you.

____
____
